# *N*-{4-[(3-Methyl­phen­yl)sulfamo­yl]phen­yl}benzamide

**DOI:** 10.1107/S1600536811040384

**Published:** 2011-10-08

**Authors:** Manu Lahtinen, Jyothi Damodara, Poornima Upadhyaya, Nonappa, Erkki Kolehmainen

**Affiliations:** a University of Jyväskylä, Department of Chemistry, PO Box 35, FI-40014 JY, Finland; b Department of Chemistry, St. Joseph Engineering College, Vamanjoor, Mangalore 575 028, India

## Abstract

In the title compound, C_20_H_18_N_2_O_3_S, the dihedral angle between the central benzene ring and the amide group is 24.1 (3)° and that between this ring and the aromatic ring of the tolyl group is 68.2 (16)°. In the crystal, adjacent mol­ecules are linked by N—H⋯O hydrogen bonds into a linear chain running along [100]. Weak C—H⋯O contacts also occur. Extensive weak π–π inter­actions exist from both face-to-face and face-to-edge inter­actions occur between the aromatic rings [centroid–centroid distances = 3.612 (2) and 4.843 (2) Å].

## Related literature

For related structures, see: Aziz-ur-Rehman *et al.* (2010*a* ▶,*b* ▶,*c* ▶); Khan *et al.* (2010 ▶); Shad *et al.* (2008 ▶, 2009 ▶); Yasmeen *et al.* (2010 ▶); Gowda *et al.* (2007 ▶).
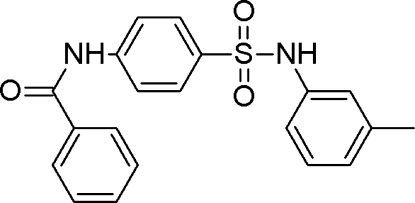


## Experimental

### 

#### Crystal data

C_20_H_18_N_2_O_3_S
*M* *_r_* = 366.42
Triclinic, 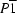

*a* = 8.5344 (2) Å
*b* = 8.8477 (3) Å
*c* = 12.4383 (4) Å
α = 77.924 (2)°
β = 75.382 (2)°
γ = 86.537 (2)°
*V* = 888.67 (5) Å^3^
*Z* = 2
Mo *K*α radiation
μ = 0.21 mm^−1^
*T* = 123 K
0.32 × 0.20 × 0.16 mm

#### Data collection

Nonius KappaCCD diffractometer with Bruker APEXII detector
Absorption correction: multi-scan (*SADABS*; Sheldrick, 1996 ▶) *T*_min_ = 0.675, *T*_max_ = 0.746
11971 measured reflections
3122 independent reflections
2591 reflections with *I* > 2σ(*I*)
*R*_int_ = 0.040

#### Refinement

*R*[*F* ^2^ > 2σ(*F* ^2^)] = 0.037
*wR*(*F* ^2^) = 0.088
*S* = 1.04
3122 reflections
242 parameters
2 restraints
H atoms treated by a mixture of independent and constrained refinement
Δρ_max_ = 0.22 e Å^−3^
Δρ_min_ = −0.41 e Å^−3^

### 

Data collection: *COLLECT* (Nonius, 1999 ▶); cell refinement: *DENZO-SMN* (Otwinowski & Minor, 1997 ▶; Otwinowski *et al.* 2003 ▶); data reduction: *DENZO-SMN*; program(s) used to solve structure: *SHELXS97* (Sheldrick, 2008 ▶); program(s) used to refine structure: *SHELXL97* (Sheldrick, 2008 ▶); molecular graphics: *Mercury* (Macrae *et al.*, 2006) ▶; software used to prepare material for publication: *WinGX* (Farrugia, 1999 ▶).

## Supplementary Material

Crystal structure: contains datablock(s) I, global. DOI: 10.1107/S1600536811040384/ng5238sup1.cif

Structure factors: contains datablock(s) I. DOI: 10.1107/S1600536811040384/ng5238Isup4.hkl

Supplementary material file. DOI: 10.1107/S1600536811040384/ng5238Isup3.cml

Additional supplementary materials:  crystallographic information; 3D view; checkCIF report

## Figures and Tables

**Table 1:** Hydrogen-bond geometry (Å, °)

*D*—H⋯*A*	*D*—H	H⋯*A*	*D*⋯*A*	*D*—H⋯*A*
N7—H7⋯O27^i^	0.86 (2)	1.99 (2)	2.813 (2)	160 (2)
N25—H25⋯O17^ii^	0.84 (2)	2.38 (2)	3.062 (2)	140 (2)
C4—H4⋯O18	0.95	2.40	3.047 (3)	125

Symmetry codes: (i) 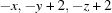; (ii) 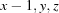.

**Fig. 1.**
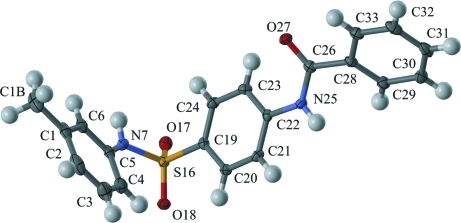
The molecular structure of title compound showing 50% propability displacement ellipsoids and the atomic numbering.

**Fig. 2.**
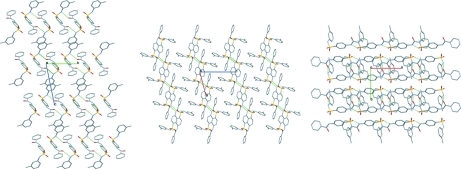
Molecular packing along a-, b- and c-axes from left to right, respectively.

**Fig. 3.**
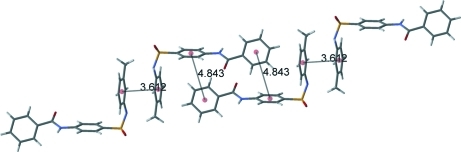
Examples of extensive π-π interaction networks between neighbouring molecules.

## supplementary crystallographic information

### Comment

Sulfonamides are very important class of compounds because of their antibacterial and enzyme inhibitor properties as well as their extensive use in medicine. As a contribution to a structural study of sulfonamide derivatives (Khan *et al.*, 2010; Aziz-ur-Rehman *et al.*, 2010*a*,*b*,*c*; Yasmeen *et al.*, 2010; Gowda *et al.* 2007), we report here the title compound, *N*-{4-[(3-methylphenyl)sulfamoyl]phenyl}benzamide (I).

Compound (I) crystallizes in triclinic space group P-1 (No. 2) without any solvent molecules and having a single molecule in an asymmetric unit (Fig. 1). The sulfonyl and amide groups show characteristic geometries (tetrahedral and planar conformation, respectively) having typical bond distances and angles for these groups (see Tables). The dihedral angles between the central phenyl group [C(19)>C(24)] and amide group N(25)—C(26)—O(27) is about 24° and the tilting of terminal groups bonded to the sulfonamide is about 111°. The molecules are packed in infinite chains along tne *a*-axis enabling the hydrogen bond network to occur *via a* axis, whereas along *c* axis the packing is more columnar forming "box"-like shapes cornered by the sulfonyl groups (Fig. 2). The infinite hydrogen bond networks, along *a* axis, occur *via* N(7)—H(7)···O(27) and N(25)—H(25)···O(17) donor-acceptors with d(D···A) bond distances of 2.813 (2) and 3.062 (2) Å in angles of about 160° and 140°, respectively. Three weaker intramolecular hydrogen bonds exist between aromatic ring H atoms (H4, H20 H23) and O atoms O(27) and O(18) having d(D···A) distances of about 2.9–3.0 Å and having fairly unfavorable contact angles varying 105–125°. In addition extensive weak π-π interactions exist in the structure as both face-to-face and face-to-edge interactions occurs between the phenyl rings (Fig. 3).

### Experimental

4-Amino-*N*-(3-methylphenyl)benzenesulfonamide (0.5 g, 1.91 mmol) was taken in 20 ml dry EtOH and then benzoyl chloride (0.22 ml, 1.91 mmol) was added dropwise. The reaction medium was maintained at basic condition by adding pyridine to neutralize the produced HCl. The mixture was refluxed at 343 K for 2 h to complete the reaction. The progress of the reaction was monitored by TLC.  A white precipitate obtained was filtered and purified in acetone to constant melting point. Few crystals suitable for a single-crystal structure determination were recrystallized from ethanol-acetone solution.

### Refinement

Hydrogen atoms were either calculated to their positions as riding atoms (C host) or taken from the electron density map (N host) using isotropic displacement parameters that were fixed to be 1.2 or 1.5 times larger than those of the attached non-hydrogen atom.

### Crystal data

**Table:**

C_20_H_18_N_2_O_3_S	*Z* = 2
*M**_r_* = 366.42	*F*(000) = 384
Triclinic, *P*1	*D*_x_ = 1.369 Mg m^−^^3^
*a* = 8.5344 (2) Å	Mo *K*α radiation, λ = 0.71073 Å
*b* = 8.8477 (3) Å	Cell parameters from 4151 reflections
*c* = 12.4383 (4) Å	θ = 0.4–28.3°
α = 77.924 (2)°	µ = 0.21 mm^−^^1^
β = 75.382 (2)°	*T* = 123 K
γ = 86.537 (2)°	Block, colourless
*V* = 888.67 (5) Å^3^	0.32 × 0.20 × 0.16 mm

### Data collection

**Table:**

Nonius KappaCCD diffractometer with Bruker APEXII detector	3122 independent reflections
Radiation source: fine-focus sealed tube	2591 reflections with *I* > 2σ(*I*)
graphite	*R*_int_ = 0.040
φ and ω scans	θ_max_ = 25.0°, θ_min_ = 2.5°
Absorption correction: multi-scan (*SADABS*; Sheldrick, 1996)	*h* = −10→10
*T*_min_ = 0.675, *T*_max_ = 0.746	*k* = −10→10
11971 measured reflections	*l* = −14→14

### Refinement

**Table:**

Refinement on *F*^2^	Primary atom site location: structure-invariant direct methods
Least-squares matrix: full	Secondary atom site location: difference Fourier map
*R*[*F*^2^ > 2σ(*F*^2^)] = 0.037	Hydrogen site location: inferred from neighbouring sites
*wR*(*F*^2^) = 0.088	H atoms treated by a mixture of independent and constrained refinement
*S* = 1.04	*w* = 1/[σ^2^(*F*_o_^2^) + (0.0225*P*)^2^ + 0.7958*P*] where *P* = (*F*_o_^2^ + 2*F*_c_^2^)/3
3122 reflections	(Δ/σ)_max_ = 0.001
242 parameters	Δρ_max_ = 0.22 e Å^−^^3^
2 restraints	Δρ_min_ = −0.41 e Å^−^^3^

### Special details

**Table:**

Geometry. All s.u.'s (except the s.u. in the dihedral angle between two l.s. planes) are estimated using the full covariance matrix. The cell s.u.'s are taken into account individually in the estimation of s.u.'s in distances, angles and torsion angles; correlations between s.u.'s in cell parameters are only used when they are defined by crystal symmetry. An approximate (isotropic) treatment of cell s.u.'s is used for estimating s.u.'s involving l.s. planes.
Refinement. Refinement of *F*^2^ against ALL reflections. The weighted *R*-factor *wR* and goodness of fit *S* are based on *F*^2^, conventional *R*-factors *R* are based on *F*, with *F* set to zero for negative *F*^2^. The threshold expression of *F*^2^ > 2σ(*F*^2^) is used only for calculating *R*-factors(gt) *etc*. and is not relevant to the choice of reflections for refinement. *R*-factors based on *F*^2^ are statistically about twice as large as those based on *F*, and *R*- factors based on ALL data will be even larger.

### Fractional atomic coordinates and isotropic or equivalent isotropic displacement parameters (Å^2^)

**Table:**

	*x*	*y*	*z*	*U*_iso_*/*U*_eq_	
C28	−0.6233 (2)	0.8118 (2)	1.16798 (16)	0.0171 (4)	
C29	−0.6766 (2)	0.6615 (2)	1.21654 (17)	0.0204 (4)	
H29	−0.6033	0.5769	1.2093	0.024*	
C30	−0.8372 (2)	0.6356 (3)	1.27566 (18)	0.0246 (5)	
H30	−0.8736	0.5333	1.3098	0.030*	
C31	−0.9443 (2)	0.7590 (3)	1.28473 (17)	0.0257 (5)	
H31	−1.0549	0.7407	1.3229	0.031*	
C32	−0.8910 (2)	0.9090 (3)	1.23850 (18)	0.0260 (5)	
H32	−0.9646	0.9933	1.2458	0.031*	
C33	−0.7305 (2)	0.9358 (2)	1.18166 (17)	0.0204 (4)	
H33	−0.6932	1.0387	1.1519	0.025*	
C26	−0.4514 (2)	0.8461 (2)	1.10486 (16)	0.0167 (4)	
C22	−0.2076 (2)	0.7276 (2)	0.99572 (16)	0.0152 (4)	
C23	−0.0888 (2)	0.7983 (2)	1.02747 (17)	0.0180 (4)	
H23	−0.1190	0.8589	1.0843	0.022*	
C24	0.0732 (2)	0.7796 (2)	0.97568 (17)	0.0176 (4)	
H24	0.1545	0.8273	0.9968	0.021*	
C19	0.1162 (2)	0.6904 (2)	0.89228 (16)	0.0155 (4)	
C20	−0.0010 (2)	0.6204 (2)	0.86079 (17)	0.0176 (4)	
H20	0.0293	0.5595	0.8042	0.021*	
C21	−0.1630 (2)	0.6395 (2)	0.91227 (16)	0.0177 (4)	
H21	−0.2439	0.5923	0.8904	0.021*	
C5	0.3326 (2)	0.9120 (2)	0.65597 (16)	0.0192 (4)	
C6	0.3642 (2)	1.0699 (2)	0.62394 (17)	0.0220 (4)	
H6	0.4185	1.1168	0.6663	0.026*	
C1	0.3176 (2)	1.1604 (3)	0.53119 (18)	0.0276 (5)	
C1B	0.3540 (3)	1.3306 (3)	0.4984 (2)	0.0390 (6)	
H1B1	0.4356	1.3519	0.4260	0.058*	
H1B2	0.3955	1.3617	0.5571	0.058*	
H1B3	0.2548	1.3890	0.4909	0.058*	
C2	0.2371 (3)	1.0896 (3)	0.47118 (19)	0.0326 (6)	
H2	0.2038	1.1492	0.4078	0.039*	
C3	0.2051 (3)	0.9333 (3)	0.50290 (19)	0.0321 (5)	
H3	0.1492	0.8870	0.4612	0.039*	
C4	0.2530 (2)	0.8425 (3)	0.59478 (17)	0.0251 (5)	
H4	0.2317	0.7347	0.6153	0.030*	
N25	−0.37393 (19)	0.73597 (19)	1.04858 (14)	0.0174 (4)	
N7	0.39203 (18)	0.82946 (19)	0.74938 (14)	0.0172 (4)	
O27	−0.38543 (16)	0.96655 (15)	1.10373 (12)	0.0208 (3)	
O17	0.41159 (15)	0.63241 (15)	0.91408 (11)	0.0196 (3)	
O18	0.33106 (15)	0.55489 (15)	0.75619 (12)	0.0204 (3)	
S16	0.32251 (5)	0.66440 (5)	0.82764 (4)	0.01601 (14)	
H7	0.415 (2)	0.887 (2)	0.7913 (17)	0.019*	
H25	−0.430 (2)	0.668 (2)	1.0390 (18)	0.019*	

### Atomic displacement parameters (Å^2^)

**Table:**

	*U*^11^	*U*^22^	*U*^33^	*U*^12^	*U*^13^	*U*^23^
C28	0.0168 (10)	0.0206 (10)	0.0157 (10)	0.0016 (8)	−0.0056 (8)	−0.0060 (8)
C29	0.0170 (10)	0.0216 (10)	0.0235 (11)	0.0017 (8)	−0.0045 (8)	−0.0075 (9)
C30	0.0206 (11)	0.0283 (12)	0.0251 (12)	−0.0053 (9)	−0.0024 (9)	−0.0081 (9)
C31	0.0157 (10)	0.0428 (13)	0.0197 (11)	−0.0004 (9)	−0.0019 (8)	−0.0114 (10)
C32	0.0205 (11)	0.0363 (13)	0.0235 (12)	0.0122 (9)	−0.0070 (9)	−0.0126 (10)
C33	0.0226 (10)	0.0221 (10)	0.0172 (10)	0.0051 (8)	−0.0064 (8)	−0.0051 (8)
C26	0.0179 (10)	0.0181 (10)	0.0147 (10)	0.0022 (8)	−0.0062 (8)	−0.0023 (8)
C22	0.0150 (9)	0.0134 (9)	0.0158 (10)	−0.0007 (7)	−0.0032 (7)	0.0000 (8)
C23	0.0201 (10)	0.0163 (10)	0.0192 (10)	0.0025 (8)	−0.0060 (8)	−0.0066 (8)
C24	0.0164 (10)	0.0160 (10)	0.0218 (11)	−0.0003 (8)	−0.0069 (8)	−0.0044 (8)
C19	0.0139 (9)	0.0161 (9)	0.0146 (10)	−0.0002 (7)	−0.0017 (7)	−0.0007 (8)
C20	0.0180 (10)	0.0172 (10)	0.0185 (10)	−0.0002 (8)	−0.0038 (8)	−0.0060 (8)
C21	0.0164 (10)	0.0169 (10)	0.0215 (11)	−0.0022 (8)	−0.0067 (8)	−0.0042 (8)
C5	0.0127 (9)	0.0245 (11)	0.0169 (10)	0.0040 (8)	−0.0001 (8)	−0.0022 (8)
C6	0.0177 (10)	0.0236 (11)	0.0201 (11)	0.0026 (8)	0.0021 (8)	−0.0033 (9)
C1	0.0200 (11)	0.0306 (12)	0.0227 (12)	0.0089 (9)	0.0048 (9)	0.0012 (9)
C1B	0.0414 (14)	0.0288 (13)	0.0329 (14)	0.0107 (11)	0.0030 (11)	0.0064 (11)
C2	0.0221 (11)	0.0484 (15)	0.0206 (12)	0.0082 (10)	−0.0036 (9)	0.0031 (10)
C3	0.0228 (11)	0.0491 (15)	0.0244 (12)	−0.0033 (10)	−0.0073 (9)	−0.0048 (11)
C4	0.0209 (11)	0.0307 (12)	0.0226 (12)	−0.0027 (9)	−0.0044 (9)	−0.0034 (9)
N25	0.0131 (8)	0.0159 (8)	0.0241 (9)	−0.0008 (7)	−0.0027 (7)	−0.0083 (7)
N7	0.0161 (8)	0.0172 (9)	0.0190 (9)	−0.0018 (7)	−0.0052 (7)	−0.0038 (7)
O27	0.0210 (7)	0.0163 (7)	0.0256 (8)	−0.0022 (6)	−0.0036 (6)	−0.0073 (6)
O17	0.0158 (7)	0.0213 (7)	0.0223 (8)	0.0018 (6)	−0.0071 (6)	−0.0032 (6)
O18	0.0183 (7)	0.0191 (7)	0.0249 (8)	0.0006 (6)	−0.0026 (6)	−0.0100 (6)
S16	0.0132 (2)	0.0155 (2)	0.0188 (3)	0.00087 (18)	−0.00303 (18)	−0.00364 (19)

### Geometric parameters (Å, °)

**Table:**

C28—C29	1.392 (3)	C20—C21	1.386 (3)
C28—C33	1.396 (3)	C20—H20	0.9500
C28—C26	1.495 (3)	C21—H21	0.9500
C29—C30	1.390 (3)	C5—C4	1.388 (3)
C29—H29	0.9500	C5—C6	1.394 (3)
C30—C31	1.385 (3)	C5—N7	1.428 (3)
C30—H30	0.9500	C6—C1	1.391 (3)
C31—C32	1.386 (3)	C6—H6	0.9500
C31—H31	0.9500	C1—C2	1.389 (3)
C32—C33	1.383 (3)	C1—C1B	1.506 (3)
C32—H32	0.9500	C1B—H1B1	0.9800
C33—H33	0.9500	C1B—H1B2	0.9800
C26—O27	1.231 (2)	C1B—H1B3	0.9800
C26—N25	1.360 (2)	C2—C3	1.380 (3)
C22—C21	1.390 (3)	C2—H2	0.9500
C22—C23	1.400 (3)	C3—C4	1.390 (3)
C22—N25	1.410 (2)	C3—H3	0.9500
C23—C24	1.386 (3)	C4—H4	0.9500
C23—H23	0.9500	N25—H25	0.835 (15)
C24—C19	1.396 (3)	N7—S16	1.6280 (16)
C24—H24	0.9500	N7—H7	0.861 (15)
C19—C20	1.383 (3)	O17—S16	1.4400 (14)
C19—S16	1.7642 (18)	O18—S16	1.4328 (14)
			
C29—C28—C33	119.77 (18)	C22—C21—H21	119.8
C29—C28—C26	121.93 (17)	C4—C5—C6	119.79 (19)
C33—C28—C26	118.25 (18)	C4—C5—N7	123.68 (18)
C30—C29—C28	119.81 (18)	C6—C5—N7	116.48 (18)
C30—C29—H29	120.1	C1—C6—C5	121.3 (2)
C28—C29—H29	120.1	C1—C6—H6	119.3
C31—C30—C29	120.0 (2)	C5—C6—H6	119.3
C31—C30—H30	120.0	C2—C1—C6	118.3 (2)
C29—C30—H30	120.0	C2—C1—C1B	121.6 (2)
C30—C31—C32	120.40 (19)	C6—C1—C1B	120.1 (2)
C30—C31—H31	119.8	C1—C1B—H1B1	109.5
C32—C31—H31	119.8	C1—C1B—H1B2	109.5
C33—C32—C31	119.89 (19)	H1B1—C1B—H1B2	109.5
C33—C32—H32	120.1	C1—C1B—H1B3	109.5
C31—C32—H32	120.1	H1B1—C1B—H1B3	109.5
C32—C33—C28	120.08 (19)	H1B2—C1B—H1B3	109.5
C32—C33—H33	120.0	C3—C2—C1	120.6 (2)
C28—C33—H33	120.0	C3—C2—H2	119.7
O27—C26—N25	122.61 (17)	C1—C2—H2	119.7
O27—C26—C28	121.90 (17)	C2—C3—C4	121.2 (2)
N25—C26—C28	115.48 (16)	C2—C3—H3	119.4
C21—C22—C23	119.99 (17)	C4—C3—H3	119.4
C21—C22—N25	117.37 (16)	C5—C4—C3	118.8 (2)
C23—C22—N25	122.59 (17)	C5—C4—H4	120.6
C24—C23—C22	119.69 (18)	C3—C4—H4	120.6
C24—C23—H23	120.2	C26—N25—C22	127.54 (16)
C22—C23—H23	120.2	C26—N25—H25	118.0 (15)
C23—C24—C19	119.67 (17)	C22—N25—H25	114.2 (15)
C23—C24—H24	120.2	C5—N7—S16	124.74 (13)
C19—C24—H24	120.2	C5—N7—H7	114.5 (14)
C20—C19—C24	120.72 (17)	S16—N7—H7	109.6 (14)
C20—C19—S16	119.57 (15)	O18—S16—O17	118.60 (8)
C24—C19—S16	119.70 (14)	O18—S16—N7	109.02 (8)
C19—C20—C21	119.61 (18)	O17—S16—N7	104.61 (8)
C19—C20—H20	120.2	O18—S16—C19	107.45 (8)
C21—C20—H20	120.2	O17—S16—C19	108.77 (8)
C20—C21—C22	120.32 (17)	N7—S16—C19	107.99 (8)
C20—C21—H21	119.8		
			
C33—C28—C29—C30	1.8 (3)	C5—C6—C1—C2	0.6 (3)
C26—C28—C29—C30	179.18 (18)	C5—C6—C1—C1B	−179.75 (18)
C28—C29—C30—C31	0.9 (3)	C6—C1—C2—C3	−0.3 (3)
C29—C30—C31—C32	−2.2 (3)	C1B—C1—C2—C3	−180.0 (2)
C30—C31—C32—C33	0.8 (3)	C1—C2—C3—C4	−0.4 (3)
C31—C32—C33—C28	1.9 (3)	C6—C5—C4—C3	−0.6 (3)
C29—C28—C33—C32	−3.2 (3)	N7—C5—C4—C3	−177.87 (18)
C26—C28—C33—C32	179.34 (18)	C2—C3—C4—C5	0.9 (3)
C29—C28—C26—O27	−148.36 (19)	O27—C26—N25—C22	9.8 (3)
C33—C28—C26—O27	29.1 (3)	C28—C26—N25—C22	−170.63 (17)
C29—C28—C26—N25	32.1 (3)	C21—C22—N25—C26	−158.59 (19)
C33—C28—C26—N25	−150.49 (18)	C23—C22—N25—C26	24.1 (3)
C21—C22—C23—C24	−0.3 (3)	C4—C5—N7—S16	−24.1 (3)
N25—C22—C23—C24	177.01 (17)	C6—C5—N7—S16	158.56 (14)
C22—C23—C24—C19	0.0 (3)	C5—N7—S16—O18	55.80 (17)
C23—C24—C19—C20	0.0 (3)	C5—N7—S16—O17	−176.39 (15)
C23—C24—C19—S16	−179.17 (14)	C5—N7—S16—C19	−60.64 (17)
C24—C19—C20—C21	0.3 (3)	C20—C19—S16—O18	−5.69 (18)
S16—C19—C20—C21	179.41 (15)	C24—C19—S16—O18	173.47 (15)
C19—C20—C21—C22	−0.5 (3)	C20—C19—S16—O17	−135.24 (15)
C23—C22—C21—C20	0.5 (3)	C24—C19—S16—O17	43.92 (18)
N25—C22—C21—C20	−176.90 (17)	C20—C19—S16—N7	111.78 (16)
C4—C5—C6—C1	−0.1 (3)	C24—C19—S16—N7	−69.07 (17)
N7—C5—C6—C1	177.35 (17)		

### Hydrogen-bond geometry (Å, °)

**Table:**

*D*—H···*A*	*D*—H	H···*A*	*D*···*A*	*D*—H···*A*
N7—H7···O27^i^	0.86 (2)	1.99 (2)	2.813 (2)	160.(2)
N25—H25···O17^ii^	0.84 (2)	2.38 (2)	3.062 (2)	140.(2)
C4—H4···O18	0.95	2.40	3.047 (3)	125
C20—H20···O18	0.95	2.49	2.884 (2)	105
C23—H23···O27	0.95	2.39	2.907 (2)	114

Symmetry codes: (i) −*x*, −*y*+2, −*z*+2; (ii) *x*−1, *y*, *z*.
